# Host Fecal mRNAs Predicted Environmental Enteric Dysfunction among Children with Moderate Acute Malnutrition in Sierra Leone

**DOI:** 10.4269/ajtmh.21-0348

**Published:** 2021-08-30

**Authors:** Akriti Singh, Isabel Potani, Stacy P. Griswold, Devika Suri, Breanne Langlois, Ye Shen, Shelley M. Walton, Kenneth Kwan Ho Chui, Mark J. Manary, Patrick Webb, Beatrice L. Rogers, Irwin H. Rosenberg

**Affiliations:** ^1^ Friedman School of Nutrition Science and Policy, Tufts University, Boston, Massachusetts;; ^2^ School of Medicine, Tufts University, Boston, Massachusetts;; ^3^ Department of Pediatrics, Washington University, St. Louis, Missouri

## Abstract

Examining the role of environmental enteric dysfunction (EED) in child growth requires noninvasive, field-appropriate biomarkers. Alternatives to the traditionally used lactulose:mannitol (L:M) test have been explored, but few studies have compared the L:M test to host fecal mRNA transcripts. The objectives of this study were to examine whether 1) host fecal mRNA transcripts could predict presence and severity of EED, measured using the L:M test, and 2) EED modifies the effect of specialized nutritious foods (SNFs) on recovery from moderate acute malnutrition (MAM). This substudy was nested within a cluster randomized trial comparing four SNFs in the treatment of MAM among children 6 to 59 months in Sierra Leone. EED was assessed at enrollment using the L:M test and 15 host fecal mRNA transcripts on 522 children. Recovery from MAM was defined as achieving mid-upper arm circumference ≥ 12.5 cm within 12 weeks of supplementation. Random forest classification models were used to examine prediction of presence and severity of EED by host fecal mRNA transcripts. Logistic regression was used to test for effect modification by L:M test variables including % lactulose excreted (%L). Eight host fecal mRNA transcripts (AQP9, REG3A, IFI30, DECR1, BIRC3, SELL, PIK3AP1, DEFA6) identified EED (%L ≥ 0.2) and severe EED (%L ≥ 0.45) with high sensitivity and specificity. The L:M test variables did not modify the effect of SNFs on recovery from MAM. In this study, we found host fecal mRNA transcripts that could be biomarkers of EED but did not find EED to modify the effect of SNFs on MAM treatment.

## INTRODUCTION

Environmental enteric dysfunction (EED), an impairment of the structure and function of the small intestine, may be involved in processes contributing to undernutrition, including moderate acute malnutrition (MAM).^1^ EED is characterized by morphologic changes including blunted villi, inflammation of the permeable intestinal walls, and poor absorption of nutrients.^2^^,^^3^ Measuring EED is challenging. Although biopsy of the small intestine is the only direct way to assess intestinal health, this invasive method is neither feasible nor ethical in most settings or in otherwise healthy subjects. For decades, the lactulose:mannitol test (L:M test) has been the most commonly used noninvasive biomarker of EED.^4^ This test comprises an oral dose of two sugars, lactulose and mannitol. The recovery of lactulose (%L) measures intestinal permeability, whereas the recovery of mannitol (%M) measures absorptive surface area. The ratio of the two sugars (L:M ratio) or the ratio of the recovery of the two sugars [L:M excretion ratio (LMER)] measures overall intestinal health. Despite being widely used, the L:M test has a number of drawbacks, such as inconvenience to participants (fasting requirements and long wait times) and inability to measure intestinal inflammation.^4^

Several alternative biomarkers to the L:M test are currently being explored.^5^ One of these is host fecal messenger ribonucleic acid (mRNA) transcripts. Studies from Malawi have shown that certain host fecal mRNA transcripts correlate with the L:M ratio and %L.^6^^–^^9^ These mRNA transcripts may be more informative than the L:M test because they capture a range of domains of EED: inflammation, permeability, repair/injury, and antimicrobial defense. Seven of these host fecal mRNA transcripts have identified severe EED (L:M ratio ≥ 0.45) among children aged 12 to 61 months, with high sensitivity and specificity.^8^ In field settings, stool samples are also relatively easier to collect than all urine voided over several hours. Notably, exploration of the host fecal mRNA transcripts in relation to the L:M test has not been conducted in settings outside Malawi or among children with acute malnutrition.

High prevalence of enteropathy has been documented among children with severe acute malnutrition (SAM), which suggests that this condition may also afflict children with MAM.^10^^–^^12^ Although many studies have examined the association between EED and linear growth, there is less evidence for an association between EED and ponderal growth.^11^^,^^13^^–^^15^ Previous studies have shown an inverse relationship between biomarkers of EED and weight-for-age z score,^11^^,^^14^ weight-for-length z score,^15^ and weight gain velocity (kilogram/month).^13^ These findings show that EED may play a role in weight gain, which is critical during MAM treatment because children must gain weight rapidly to recover from their high mortality risk, undernourished state. Specialized nutritious foods (SNFs) have been used for many years to treat children with MAM. However, the biological pathways by which these foods enable recovery from MAM is poorly understood, which affects product optimization and tailoring of complementary interventions.^16^

Therefore, the objectives of this study were to examine whether 1) host fecal mRNA transcripts could predict the presence and severity of EED measured using the L:M test and 2) EED modifies the effect of SNFs on recovery from MAM. Results from this study will add to the evidence base for an alternative biomarker of EED and the role of EED in children with MAM.

## MATERIALS AND METHODS

### Study design.

This study was nested within the Four Foods MAM Treatment Study conducted in the Pujehun district of Sierra Leone from April 2017 and November 2018. The Four Foods MAM Treatment Study was a cluster-randomized, clinical and cost-effectiveness trial assessing four SNFs to treat children 6 to 59 months with MAM, defined as mid-upper arm circumference (MUAC) ≥ 11.5 cm and < 12.5 cm without bipedal edema. Details of the parent study have been described previously.^17^ The four foods were 1) corn soy blend plus with fortified vegetable oil (CSB+ w/oil), serving as the reference group; 2) super cereal plus with amylase (SC+A); 3) corn soy whey blend with fortified vegetable oil (CSWB w/oil); and 4) a lipid-based ready-to-use supplementary food (RUSF).

Participants were eligible to receive the study arm food until they reached a MUAC of ≥ 12.5 cm or for up to 12 weeks, whichever occurred sooner.

The Four Foods MAM Treatment Study was conducted in 29 peripheral health units (PHUs) randomly assigned to deliver one of four isocaloric foods. This study was conducted at eight of the study PHUs, two per arm, purposively selected based on feasibility. Biological samples were collected at enrollment into the MAM treatment program from July 2017 to August 2018.

### Approvals.

The study was approved by the Sierra Leone Ethics and Scientific Review Committee and the Tufts University Health Sciences Institutional Review Board. Written informed consent was obtained from caregivers of all study participants.

### Sample size.

The sample size was based on the secondary objective because data comparing host fecal mRNA transcripts to L:M ratio among children with acute malnutrition are limited. To examine whether EED modifies the effect of SNFs on recovery from MAM, the total planned sample size was 404 (100 per arm), which achieves 80% power to detect an R^2^ value of 0.2 in a multivariable regression model with eight predictors at a significance level (alpha) of 0.05, assuming a design effect of 1.2.

### L:M test.

Details of sample collection have been described previously.^18^ At the PHU, a 20-mL dose comprising 5 g of lactulose (McKesson, San Francisco, CA) and 1 g of mannitol (Sigma Aldrich, St. Louis, MO) was orally given to each participant, using a medicine cup or syringe, after an 8-hour overnight fast. Water was allowed as often as desired by the child before and after being dosed with the sugar solution. After dosing, a urine collection bag (Thermo Fisher Scientific, Waltham, MA) and a locally prepared, nonabsorbent diaper were attached to the participant.

Urine excreted over the next 4 hours was collected in a cup with 10 mg Thimerosal (Sigma Aldrich). The urine was mixed with a pipette, and a 3-mL aliquot was transferred to plastic cryovials, and flash frozen in liquid nitrogen at the PHU. Participants and caregivers were provided lunch 3 hours after dose administration. At this time, breastfeeding was also allowed. The total urine volume was recorded using a graduated cylinder. Every month, samples were transferred from the liquid nitrogen tanks to a –20°C freezer at University of Makeni before being shipped on dry ice to Baylor College of Medicine, Houston, TX. The concentration of the sugars in the urine was analyzed via high performance liquid chromatography.^19^

Concentration of the two sugars in the samples collected were used to calculate the L:M test variables. Percentage lactulose excreted (%L) was calculated as the concentration of lactulose in urine sample multiplied by the 4-hour volume of urine and divided by the concentration of lactulose in the dose. Percentage mannitol excreted (%M) was calculated similarly. L:M excretion ratio (LMER) was calculated as the percentage of lactulose excreted divided by the percentage of mannitol excreted. The L:M ratio was calculated as the concentration of lactulose divided by the concentration of mannitol in the urine sample. The concentration of L and M was detected in all collected urine samples.

Although lactulose: mannitol excretion ratio (LMER) or L:M ratio have historically been the markers of EED, new evidence suggests that both high or low LMER or L:M ratio could represent poor gut function and that %L may be a more accurate measure of intestinal health.^20^ For this reason, our primary L:M test indicator was %L, but we also conducted the analysis with LMER and L:M ratio. Severity terciles of %L were generated based on existing literature.^8^ No EED as %L < 0.2, medium as %L 0.2 to < 0.45, and high EED as %L ≥ 0.45 based on associations with linear growth.^7^

### Host fecal mRNA transcripts.

Stool samples were collected at any point before, during, or after the 4-hour wait period for the L:M test. Once a participant had a bowel movement, the diaper was removed, all stool collected was mixed with a spatula, and aliquots were transferred into plastic cryovials without fixative. The cryovials with stool were flash-frozen in liquid nitrogen at the PHU. Every month, samples were transferred from the liquid nitrogen tanks to a –80°C freezer at University of Makeni before being shipped on dry ice to Washington University at St. Louis School of Medicine (St. Louis, MO) for analysis. The 15 host fecal mRNA transcripts, previously reported to correlate with the L:M ratio, were analyzed by digital droplet polymerase chain reaction (PCR).^8^^,^^21^

The host fecal mRNAs were extracted from stool samples using NucliSENS easyMAG system (bioMerieux, Durham, NC) as per the protocol described by Stauber et al.^21^ The extracted mRNA transcripts were assayed in a droplet digital PCR system (QX100; Bio-Rad Laboratories, Inc., Hercules, CA). These mRNA transcripts measured inflammation (AQP91, CD53, LYZ, IFI30, PIK3AP1, S100A8, SELL), structural integrity (BIRC3, CDX1, MUC12, DECR1, HLA-DRA), and repair/antimicrobial defense (REG3A, DEFA6, and REG1A). The function of each has been described previously along with their correlation with the L:M test variables.^18^ The copies of the mRNA transcripts were normalized to glyceraldehyde 3-phosphate dehydrogenase (GAPDH). Samples with GAPDH < 25 copies could not be used to assay the mRNA transcript and were treated as zero.

### Statistical analysis.

Statistical analyses were conducted using Stata 15 software (StataCorp, College Station, TX). Distribution of the biomarkers was examined for outliers and normality. One outlier for the host fecal mRNA transcript, lysozyme (LYZ), was excluded from analysis because it was 140 times higher than values for other participants. All biomarkers were natural log (ln) transformed, as they were right skewed. Background characteristics of study participants were examined overall and by study arm. The primary outcome was %L, and the secondary outcome was recovery from MAM within 12 weeks of supplementation.

Two random forest classification models were estimated using the Stata ‘randomforest’ module to assess the most important host fecal mRNA transcripts that predicted EED using %L, and how well the important predictors could identify children with EED. Two separate outcomes were assessed: 1) presence (%L ≥ 0.2) versus absence (%L < 0.2) of EED and 2) severity of EED: none (%L < 0.2), moderate (%L 0.2 to < 0.45), or high (%L ≥ 0.45). The average prediction error was measured using the out-of-bag error rate (number of incorrect predictions/number of iterations where the observation was not in the training set) for each model. Each model included 1,000 iterations. Test characteristics were calculated using the predicted classes.

Recovery from MAM was defined as achieving a MUAC ≥ 12.5 cm within 12 weeks of supplementation. The L:M test variables were %L, %M, LMER, and L:M ratio. Logistic regression was used to examine difference in recovery from MAM by study arm controlling for the L:M test markers. Then an interaction term for the L:M test markers and study arm was added to the model to assess effect modification. Adjusted models also controlled for covariates selected based on biological relevance (age, sex, and previous SAM status of the child). Covariates were extracted from the Four Foods MAM Treatment Study. Pregibon’s delta beta statistics for influential observations, and Hosmer-Lemeshow goodness-of-fit test were performed. The standard errors were not adjusted for PHU level clustering because mixed models with the interaction terms controlled for clustering were unstable, likely due to the small number of clusters (*N* = 8). Statistical significance was set at *P* value < 0.05 for all analyses.

## RESULTS

### Study population.

**Figure 1.**
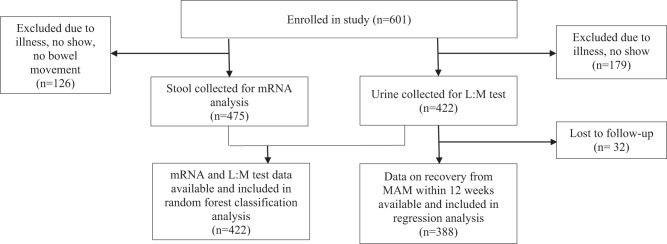
Participant flow diagram.

Figure 1 shows the participant flow diagram. Table 1 presents characteristics of 422 study participants that contributed to the L:M test marker results. At enrollment, the participants’ mean age ± SD was 14.52 ± 8.93 months, 55% were female, and 23% had transferred from an SAM treatment program. The mean ± SD for MUAC was 11.97 ± 0.27, length-for-age z score (LAZ) was –2.75 ± 1.25, and weight-for-length z score (WLZ) was –1.89 ± 0.74. Overall, 68% of study participants graduated from the treatment program within 12 weeks. These characteristics were balanced across arm except for transfer from SAM.

**Table 1:** Characteristics of study participants at enrollment and recovery within 12 weeks of treatment

	All	CSWB with oil	SC+A	CSB+ w/ oil	RUSF	*P* value*
*n*	422	49	121	98	144	
Age (months)	14.52 ± 8.93	13.24 ± 9.13	14.21 ± 9.32	14.32 ± 8.11	15.42 ± 9.05	0.421
Female	233 (55%)	34 (59%)	74 (61%)	45 (46%)	80 (56%)	0.168
Transferred from SAM	95 (23%)	12 (21%)	27 (22%)	32 (32%)	24 (17%)	0.029†
Anthropometry						
MUAC	11.97 ± 0.27	12.01 ± 0.28	11.95 ± 0.26	11.96 ± 0.26	11.98 ± 0.26	0.518
LAZ	−2.75 ± 1.25	−2.69 ± 1.21	−2.71 ± 1.26	−2.82 ± 1.29	−2.77 ± 1.24	0.897
WLZ	−1.89 ± 0.74	−1.72 ± 0.79	−1.83 ± 0.73	−1.92 ± 0.75	−1.99 ± 0.7	0.081
Recovery (*N* = 484)	327 (68%)	48 (65%)	93 (65%)	78 (64%)	108 (74%)	0.165

CSB = corn soy blend; CSWB = corn soy whey blend; LAZ = length-for-age z score; MUAC = mid-upper arm circumference; RUSF = ready-to-use supplementary food; SAM = severe acute malnutrition; SC+A = super cereal plus with amylase; WLZ, weight-for-length z score. Cells represent mean ± SD or n (%).

* *P* value for difference between SNFs by linear regression for continuous variables, χ^2^ test for categorical variables.

† *P* < 0.05.

### EED biomarkers.

Table 2 shows enrollment biomarker values. The majority (77%) of the study participants had EED (%L ≥ 0.2) at enrollment. The median (interquartile range) for LMER, L:M ratio, %L, and %M were 0.09 (0.06–0.15), 0.47 (0.31–0.73), 0.34 (0.21–0.61), and 3.87 (2.46–5.67). No cutoffs have been established for the host fecal mRNA transcripts.

**Table 2:** Environmental enteric dysfunction biomarkers at enrollment

	*n*	Median (25th, 75th percentile)
Lactulose:mannitol (L:M) test		
%L	422	0.34 (0.21, 0.61)
%M	422	3.87 (2.46, 5.67)
L:M excretion ratio	422	0.09 (0.06, 0.15)
L:M ratio	422	0.47 (0.31, 0.73)
Fecal host mRNA transcripts*		
AQP9	427	0.13 (0.06, 0.31)
BIRC3	428	0.22 (0.11, 0.37)
CD53	434	0.20 (0.07, 0.55)
CDX1	435	0.05 (0.03, 0.08)
DECR1	429	0.06 (0.03, 0.09)
DEFA6	429	0.08 (0.04, 0.21)
HLA-DRA	433	0.14 (0.07, 0.24)
IFI30	429	0.31 (0.14, 0.65)
LYZ	441	0.11 (0.05, 0.21)
MUC12	435	0.44 (0.23, 0.84)
PIK3AP1	429	0.15 (0.06, 0.39)
REG1A	435	0.14 (0.06, 0.35)
REG3A	429	0.07 (0.03, 0.15)
S100A8	433	1.19 (0.53, 2.81)
SELL	429	0.06 (0.02, 0.16)

AQP9 = aquaporin 9; BIRC3 = Baculoviral Inhibitor of Apoptosis Repeat Containing 3; CD53 = cluster of differentiation 53; CDX1 = caudal type homeobox 1; DECR1 = 2, 4-dienoyl-coa reductase 1; DEFA6 = defensin alpha 6; HLA-DRA = major histocompatibility complex class II DR alpha; IFI30 = gamma-interferon-inducible lysosomal thiol reductase; LYZ = lysozyme; MUC12 = mucin 12; PIK3AP1 = phosphoinositide-3-kinase adaptor protein 1; REG1A = regenerating islet-derived 1 alpha; REG3A = regenerating islet-derived 2 alpha; S100A8 = S100 calcium binding protein A8; SELL = selectin.

* Copies per copy of glyceraldehyde 3-phosphate dehydrogenase.

### Prediction of EED by mRNA transcripts.

Tables 3 and 4 show results from the random forest classification models. We found that eight host fecal mRNA transcripts (AQP9, REG3A, IFI30, DECR1, BIRC3, SELL, PIK3AP1, and DEFA6) were important predictors of %L ≥ 0.2. Furthermore, a model with these eight host fecal mRNA transcripts was able to identify %L ≥ 0.2 with 100% sensitivity and 80% specificity. These same host fecal mRNA transcripts were also able to identify all children with %L 0.2 to < 0.45 (moderate EED), and %L ≥ 0.45 (severe EED) with 84% sensitivity and 80% specificity.

**Table 3:** Sensitivity and specificity of eight host fecal mRNA transcripts to identify children with and without environmental enteric dysfunction based on random forest classification models

Predicted class	%L < 0.2, *n* (%)	%L ≥ 0.2, *n* (%)	Total, *n* (%)
No EED	74 (79.57%)*	0 (0.00%)	74 (17.54%)
EED	19 (20.43%)	329 (100.00%)†	348 (82.46%)
Total	93 (100.00%)	329 (100.00%)	422 (100.00%)

EED = environmental enteric dysfunction; %L = percent lactulose; mRNA = messenger ribonucleic acid.

* Specificity.

† Sensitivity (presence).

**Table 4:** Sensitivity and specificity of eight host fecal mRNA transcripts to identify children with no, moderate, and severe EED based on random forest classification models

Predicted class	%L < 0.2, *n* (%)	%L ≤ 0.2 to < 0.45, *n* (%)	%L ≥ 0.45, *n* (%)	Total, *n* (%)
No EED	74 (79.57%)*	0 (0.00%)	0 (0.00%)	74 (17.54%)
Moderate EED	19 (20.43%)	175 (100.00%)†	25 (16.23%)	219 (51.90%)
Severe EED	0 (0.00%)	0 (0.00%)	129 (83.77%)‡	129 (30.57%)
Total	93 (100.00%)	175 (100.00%)	154 (100.00%)	422 (100.00%)

EED = environmental enteric dysfunction; %L = percent lactulose; mRNA = messenger ribonucleic acid

* Specificity.

† Sensitivity (moderate).

‡ Sensitivity (severe).

### Recovery, study arm, and EED.

Previously, we showed that the mRNA transcripts were variably correlated with the L:M test variables.^18^ Table 5 presents the relationship between recovery from MAM, study arm, and EED at enrollment using unadjusted and adjusted logistic regression models. There was no association between study arm and recovery from MAM even after controlling for EED at enrollment using %L, %M, LMER, or LM ratio in unadjusted models (*P*_testparm_ = 0.155, 0.204, 0.160, and 0.165, respectively) and models adjusted for age, sex, and transfer from SAM (*P*_testparm_ = 0.328, 0.349, 0.357, and 0.371 respectively). Additionally, none of the L:M test markers was associated with recovery except for %M in the unadjusted model (*P* = 0.048).

**Table 5:** Effect of SNFs on recovery from MAM controlling for EED at enrollment

	Unadjusted adjusted							
	%L	%M	LMER	LM ratio	%L	%M	LMER	LM ratio
	β (95% CI)	β (95% CI)	β (95% CI)	β (95% CI)	β (95% CI)	β (95% CI)	β (95% CI)	β (95% CI)
*n*	388	388	388	388	387	387	387	387
SNFs								
CSB+ w/ oil	Reference	Reference	Reference	Reference	Reference	Reference	Reference	Reference
CSWB w/ oil	−0.19 (−0.87 to 0.50)	−0.20 (−0.88 to 0.49)	−0.18 (−0.86 to 0.51)	−0.18 (−0.86 to 0.51)	−0.27 (−1.01 to 0.47)	−0.28 (−1.02 to 0.47)	−0.28 (−1.02 to 0.47)	−0.28 (−1.02 to 0.46)
SC+A	−0.17 (−0.74 to 0.39)	−0.15 (−0.72 to 0.41)	−0.15 (−0.72 to 0.41)	−0.15 (−0.71 to 0.42)	−0.17 (−0.79 to 0.44)	−0.18 (−0.79 to 0.43)	−0.16 (−0.77 to 0.46)	−0.16 (−0.77 to 0.46)
RUSF	0.41 (−0.17 to 0.99)	0.39 (−0.20 to 0.97)	0.42 (−0.16 to 1.00)	0.42 (−0.16 to 1.00)	0.30 (−0.33 to 0.93)	0.28 (−0.35 to 0.91)	0.29 (−0.34 to 0.92)	0.28 (−0.35 to 0.91)
EED*	0.82 (−0.12 to 1.76)	0.42 (0.00 to 0.84)†	0.60 (−2.27 to 3.46)	0.11 (−0.8 to 1.02)	−0.01 (−1.02 to 1.01)	0.24 (−0.20 to 0.69)	−1.03 (−4.02 to 1.95)	−0.49 (−1.47 to 0.48)
*P*_testparm SNF_	0.155	0.204	0.160	0.165	0.328	0.349	0.357	0.371
*R*^2^	0.017	0.018	0.011	0.011	0.104	0.107	0.105	0.106

CI = confidence interval; CSB+ w/ oil = corn soy blend plus with fortified vegetable oil; CSWB w/ oil = corn soy whey blend with fortified vegetable oil; EED = environmental enteric dysfunction; L:M = lactulose:mannitol; LMER = lactulose:mannitol excretion ratio; %L = percent lactulose; %M = percent mannitol; MAM = moderate acute malnutrition; RUSF = ready-to-use supplementary food; SC+A = super cereal plus with amylase; SNFs = specialized nutritious foods. Logistic regression models adjusted for child age, gender, and previous severe acute malnutrition status. %L, %M, LMER, and LM ratio ln transformed before analysis.

* Takes the value %L, %M, LMER, and LM ratio in separate models.

† *P* < 0.05.

Table 6 shows the interaction between study arm and EED using unadjusted and adjusted logistic regression model to examine effect modification by EED. EED did not modify the effect of the study arm on recovery from MAM using %L, %M, LMER, or LM ratio in unadjusted models (*P*_interaction_ = 0.127, 0.194, 0.177, and 0.l60 respectively) or models adjusted for age, sex, and transfer from SAM (*P*_interaction_ = 0.267, 0.314, 0.082, and 0.090 respectively). The interaction term in the adjusted model was borderline significant for LMER (*P*_interaction_ = 0.082) and LM ratio (*P*_interaction_ = 0.090).

**Table 6:** Interaction between EED (as defined by %L, %M, LMER, and LM ratio), and SNFs in predicting recovery from MAM

	Unadjusted				Adjusted			
	%L	%M	LMER	LM ratio	%L	%M	LMER	LM ratio
	β (95% CI)	β (95% CI)	β (95% CI)	β (95% CI)	β (95% CI)	β (95% CI)	β (95% CI)	β (95% CI)
*n*	388	388	388	388	387	387	387	387
SNFs								
CSB+ w/ oil	Reference	Reference	Reference	Reference	Reference	Reference	Reference	Reference
CSWB w/ oil	−1.49 (−2.84 to −0.14)	−2.90 (−5.43 to −0.36)	−0.92 (−2.51 to 0.66)	−1.08 (−2.91 to 0.76)	−1.44 (−2.88 to 0.00)	−2.64 (−5.32 to 0.04)	−0.76 (−2.50 to 0.98)	−0.87 (−2.88 to 1.14)
SC+A	−0.99 (−2.06 to 0.07)	−0.59 (−2.34 to 1.15)	−1.27 (−2.42 to −0.12)	−1.52 (−2.86 to −0.19)	−0.87 (−2.00 to 0.27)	−0.38 (−2.23 to 1.47)	−1.47 (−2.69 to −0.25)	−1.73 (−3.15 to −0.31)
RUSF	−0.13 (−1.21 to 0.95)	−0.18 (−2.03 to 1.67)	−0.29 (−1.48 to 0.90)	−0.35 (−1.73 to 1.03)	−0.14 (−1.28 to 0.99)	0.03 (−1.91 to 1.97)	−0.49 (−1.77 to 0.78)	−0.56 (−2.03 to 0.92)
EED*	−0.95 (−2.87 to 0.98)	0.01 (−0.82 to 0.85)	−6.50 (−13.95 to 0.95)	−1.90 (−4.09 to 0.29)	−1.55 (−3.70 to 0.60)	−0.03 (−0.94 to 0.88)	−9.49 (−17.75 to −1.23)	−2.80 (−5.22 to −0.38)
SNFs × EED*								
CSB+ w/ oil	Reference	Reference	Reference	Reference	Reference	Reference	Reference	Reference
CSWB w/ oil	3.89 (0.35 to 7.44)	1.81 (0.16 to 3.45)	6.65 (−6.73 to 20.02)	2.00 (−1.91 to 5.91)	3.53 (−0.25 to 7.31)	1.59 (−0.15 to 3.33)	4.05 (−10.88 to 18.98)	1.25 (−3.07 to 5.57)
SC+A	2.35 (−0.18 to 4.89)	0.30 (−0.80 to 1.39)	9.79 (1.09 to 18.49)	3.01 (0.38 to 5.64)	2.03 (−0.70 to 4.75)	0.14 (−1.03 to 1.30)	11.50 (2.26 to 20.74)	3.44 (0.65 to 6.24)
RUSF	1.56 (−1.04 to 4.17)	0.38 (−0.77 to 1.53)	6.30 (−3.11 to 15.71)	1.70 (−1.12 to 4.51)	1.32 (−1.44 to 4.08)	0.17 (−1.03 to 1.38)	7.00 (−3.10 to 17.10)	1.84 (−1.17 to 4.86)
*P*_interaction_	0.127	0.194	0.177	0.160	0.267	0.314	0.082	0.090
*R*^2^	0.028	0.029	0.021	0.021	0.113	0.115	0.120	0.120

CI = confidence interval; CSB+ w/ oil = corn soy blend plus with fortified vegetable oil; CSWB w/ oil = corn soy whey blend with fortified vegetable oil; EED = environmental enteric dysfunction; L:M = lactulose:mannitol; LMER = lactulose:mannitol excretion ratio; %L = percent lactulose; %M = percent mannitol; MAM = moderate acute malnutrition; RUSF = ready-to-use supplementary food; SC+A = super cereal plus with amylase; SNFs = specialized nutritious foods.

* Takes the value %L, %M, LMER, and LM ratio in separate models.

## DISCUSSION

In this study of MAM children enrolled in a supplementary feeding program in Sierra Leone, prevalence of EED (%L) was high at 77%. We also found eight host fecal mRNA transcripts that identified presence of EED (%L ≥ 0.2) and severe EED (%L ≥ 0.45) with high sensitivity and specificity. However, there was no effect modification by EED; that is, no one food performed better at high or low levels of EED compared with CSB+ w/oil, the reference group.

We found a panel of eight host fecal mRNA transcripts that may be biomarkers of EED. In a previous study conducted among 12- to 61-month-old children at risk of stunting in Malawi, six host fecal mRNA transcripts (CDX1, HLA-DRA, MUC12, REG1A, S100A8, and TNF) were able to identify severe EED (%L ≥ 0.45) with 84% sensitivity and 73% specificity.^8^ In the same study, a separate model with four host fecal mRNA transcripts (TNF, HLA-DRA, MUC12, and CD53) was able to identify severe EED, measured using L:M ratio, with 84% sensitivity and no EED (L:M ratio ≤ 0.15) with 83% sensitivity.^8^ However, another study conducted in the same setting found that these seven host fecal mRNA transcripts (CDX1, HLA-DRA, MUC12, REG1A, S100A8, TNF, and CD53) could not predict severe EED, measured using %L, among children aged 6 to 12 months with high sensitivity and specificity.^9^ Surprisingly, none of the transcripts identified by our study was part of the panel previously found to predict severe EED. This could be due to differences in the age and/or nutrition status of the study participants. Additionally, it could also be due to variation in the causes of EED in different populations.

In a separate analysis, our group showed that EED biomarkers developed using factor analysis on the same 15 host fecal mRNA transcripts resulted in three scores measuring intestinal inflammation, permeability, and antimicrobial defense.^18^ However, those scores were not associated with the L:M test markers. This may be because the factor analysis-based scores separately identified a particular domain of EED, whereas these eight transcripts together likely capture several domains of EED.

Other studies have also found that the level of EED at enrollment does not modify the effect of a nutrition intervention. A zinc supplementation trial in Bangladesh did not find zinc absorption to vary by L:M ratio (L:M ratio < 0.90 versus ≥ 0.90).^22^ Similarly, a study from Laos also did not find that baseline EED (assessed using fecal biomarkers) modified the effect of different strategies to deliver supplemental zinc on stunting.^23^ We may not have found a role for EED in recovery from MAM for a few reasons. First, EED was only measured at enrollment but could change over the course of treatment; such month-to-month variation in L:M ratio for the same individual has been documented in Zambia.^24^ Second, the EED biomarker selected (L:M test) may not be a sensitive indicator for this kind of analysis. Third, because the foods behaved comparably in treating children with EED, it is possible that the composition of all four foods included ingredients that enabled recovery in the presence of EED.

There are limitations to our findings. Although the L:M test markers have been the most widely used measure of EED, they are not a replacement for biopsy. Because few studies have validated the L:M test against biopsies, it is possible that we may not be measuring EED with the L:M test. Similarly, the cutoff for presence of EED based on %L was not determined by biopsy but on the association with linear growth, which is a consequence of EED.

In conclusion, the finding that eight host fecal mRNA transcripts could predict presence and severity of intestinal permeability suggests that these are potential biomarkers of EED among malnourished children. Future studies should examine whether the same eight host fecal mRNA transcripts can predict EED and severity of EED among children aged 6 to 59 months of age in other low- and middle-income settings.
